# Emoji can facilitate recognition of conveyed indirect meaning

**DOI:** 10.1371/journal.pone.0232361

**Published:** 2020-04-30

**Authors:** Thomas Holtgraves, Caleb Robinson

**Affiliations:** Ball State University, Muncie, Indiana, United States of America; Florida State University, UNITED STATES

## Abstract

In face-to-face communication there are multiple paralinguistic and gestural features that facilitate recognition of a speaker’s intended meaning, features that are lacking when people communicate digitally (e.g., texting). As a result, substitutes have emerged (expressive punctuation, capitalization, etc.) to facilitate communication in these situations. However, little is known about the comprehension processes involved in digital communication. In this research we examined the role of emoji in the comprehension of face-threatening, indirect replies. Participants in two experiments read question–reply sequences and then judged the accuracy of interpretations of the replies. On critical trials the reply violated the relation maxim and conveyed a negative, face-threatening response. On one-third of the trials the reply contained only text, on one-third of the trials the reply contained text and an emoji, and on one-third of the trials the reply contained only an emoji. When the question requested potentially negative information about one of the interactants (disclosures and opinions), participants were more likely to endorse the indirect meaning of the reply, and did so faster, when the reply contained an emoji than when it did not. This effect did not occur when the question was a request for action, a more conventional type of indirect reply. Overall, then, this research demonstrates that emoji can sometimes facilitate the comprehension of meaning. Future research is needed to examine the boundary conditions for this effect.

In face-to-face communication there are multiple paralinguistic and gestural features that facilitate recognition of a speaker’s intended meaning. Recent research, for example, demonstrates how both facial expressions [1] and prosody [2] play an important role in the successful recognition of a speaker’s intention. Digital communication is lacking in such features and this may increase the likelihood of miscommunication in these modalities. And in fact, people do report higher levels of miscommunication when texting, relative to face-to-face interactions or phone calls [3,4]. The use of shortened expressions (e.g., LOL) and emblems such as emoticons and emoji have emerged as substitutes for the nonverbal behaviors that occur in face-to-face communication [5,6]. Yet their role in communication is not well understood, especially their role in the disambiguation of speaker meaning. The purpose of this research was to explore the possibility that the use of emoji may facilitate recognition of a speaker’s indirect meaning.

## Emoticon and emoji

Emoticons, or text-based representation of a face, emerged relatively early as a means of signaling that a message posted on a message board was to be taken seriously (or not). Emoji (as used in digital communication) are a more recent development and are actually pictographs (i.e., a pictorial sign or symbol as with: Fig A in S1 File). Hence, what an emoji can look like is not limited to the characters on a keyboard, but instead can depict a myriad of different facial expressions with varying levels of detail (e.g., sweat rolling down one’s face, reddened cheeks, bulging eyes). Additionally, because of their pictorial nature, emoji are not just limited to faces; emoji exist resembling an array of different objects (e.g., national flags, vehicles, various foods), animals, hand gestures (e.g., thumbs up, praying), and behaviors (e.g., dancing, running).

One popular view of emoticons is that they are used primarily to convey affective meaning [5,6,7,8,9]. Consistent with this, emoticons have been found to influence the perceived valence of text messages. For example, Derks, Bos, and von Grumbkow [5] found that participants rated messages that included emoticons as more intense than the same messages without emoticons. Other research suggests that when the valence of an emoticon is incongruent with the valence of the text, the message will be interpreted as ambiguous [10] and this can sometimes result in the sender’s message being perceived as sarcastic [8]. Kavenagh [11] examined the possibility that emoticons might sometimes be used as politeness markers. In a study of Japanese and U.S. bloggers, he found that emoticons were used by bloggers from both countries (though significantly more by the former than the latter), and could function as both a positive politeness (enhance closeness) and negative politeness (lessen imposition) marker [12].

Other scholars have argued that emoticons are not just affective, but also can serve a more pragmatic function and act as illocutionary force indicating devices thereby helping to disambiguate the sender’s intention [13]. Some research supports this idea. For example, Thompson and Filik [14] asked participants to attempt to make their intentions clear in a texting situation, either by making changes to a text message (Experiment 1) or creating their own message. Of interest was the use of emoticons. In both experiments, participants were significantly more likely to use emoticons when the intent was sarcastic rather than literal.

In a related study, Filik and Thompson [15] examined perceptions of literal and sarcastic messages as a function of whether the message included emoticons, various types of punctuation, or neither. The interpretation of an intended sarcastic message was not impacted by emoticons or punctuation when there was contextual support and the meaning clear. However, intended literal messages were more likely to be perceived as sarcastic when the message included emoticons. In contrast, when the intended meaning was ambiguous, including an emoticon (wink emoticon) increased the likelihood of sarcastic intent being recognized. Research conducted by Riordan [16, 17] suggests that these effects are not limited to face emoji. In several studies she demonstrated that the inclusion of nonface emoji in a message can reduce perceived ambiguity, and increase interpretation confidence, in addition to influencing judgments of conveyed affect, especially positive affect.

More recently, Weissman and Tanner [18] demonstrated that emojis (wink) used to convey irony generally elicit the same neural responses as irony conveyed with words. Specifically, they observed enhanced P200s (an early indicator of attention-related processes) and P600s (reflecting reanalysis or integration processes) to wink emoji. Importantly, judgments of irony correlated with the occurrence of a P600; i.e., participants who interpreted the message as ironic were more likely to display a P600. There does appear to be a processing cost associated with emoji, at least in some situations. Cohen et al. [19] examined comprehension processes when emoji were substituted for words. They examined both nouns (e.g., Fig A in S1 File for pizza) and verbs (e.g., Fig B in S1 File for love). They found a processing cost for the use of emoji (for both nouns and verbs) as indexed with self-paced reading times, a finding consistent with the enhanced P600 reported by Weissman and Tanner [18]. Interestingly, they found no differences between words and their emoji substitutes in terms of their comprehensibility.

## Indirect replies

If emoji are communicative and (sometimes) designed to disambiguate a speaker’s intended meaning, it seems that this would be most likely to occur when the intended meaning is ambiguous or indirect. There are multiple types of indirect meaning, although research has focused primarily on metaphor, sarcasm, and irony. In prior research [20, 21] we investigated the comprehension of potentially face-threatening indirect replies, a more ambiguous type of indirect meaning. Indirect replies are responses to questions in which the speaker does not provide a direct answer, and in so doing violates the Gricean [22] maxim of relation (make your contribution relevant to the exchange). For example, when Mark replies “It’s hard to give a good presentation” in response to Nick’s question “What did you think of my presentation?” observers typically reason that Mark thought poorly of Nick’s presentation and is conveying that negative opinion indirectly. Research suggests that the generation of this implicature is relatively time-consuming, and involves the activation and subsequent rejection of the literal meaning of the reply [20,21]. In Gricean terms this would be considered a particularized implicature; i.e., the interpretation of the intended meaning is entirely context dependent. Recent research suggests that comprehension of this type of indirect reply involves recruitment of brain areas involved in empathy (Right Anterior Cingulate Cortex) and inferencing (Right Superior Temporal Gyrus) [23].

As particularized implicatures, the interpretation of indirect replies requires attention to contextual cues that provide support for the intended indirect meaning. Linguistic cues can serve this function and one important class of terms in this regard is dispreferred markers. Dispreferred turn markers can take the form of delays and hesitations (‘hehh’, silent pauses), space takers (‘well’, ‘you know’), or more specific forms, like token agreements before contradiction (‘yes, but… ‘). These markers typically occur at the beginning of a turn and indicate that the present contribution is the opposite of what would be ideally expected in a cooperative interaction, that is, the turn is dispreferred in some sense; e.g., disagreeing, rejecting, blaming [24,25,26].

Research suggests that dispreferred markers can influence the interpretation of scalar expressions (e.g., some, sometimes, etc.), words that are ambiguous and interpreted differently as a function of the context [27]. More importantly, our prior research demonstrated how the discourse marker “well” can influence the comprehension of indirect replies [28]. Participants in these studies read brief question–indirect reply sequences and then indicated how the reply should be interpreted. On critical trials the interpretation following the reply was an indirect, face-threatening interpretation and the speed with which this interpretation was endorsed served as the primary dependent measure. Verification of the indirect meaning was significantly faster on the “well” trials relative to the trials in which the reply did not contain the “well” preface. Hence, the presence of the dispreferred marker “well” facilitated recognition of the speaker’s intended indirect meaning.

## The present research

The purpose of the present research was to examine the role played by emoji in the processing of potentially face-threatening indirect replies. To do this, we modified the procedure used by Holtgraves [28] and substituted emoji for the dispreferred marker “well”. Participants read situation descriptions followed by a question and reply to that question. The reply either contained an emoji or did not, and the emoji was presented either at the beginning of the reply (Experiment 1) or at the end of the reply (Experiment 2). After reading the reply, participants were asked to indicate their judgment (Yes or No) of whether the paraphrase was a reasonable interpretation of the preceding reply; on critical trials the paraphrase was a face-threatening indirect interpretation. Participants endorsement of that interpretation, and the speed with which they endorsed it, served as dependent measures. We reasoned that emoji could function similarly to dispreferred markers and facilitate recognition of the intended indirect meaning. Hence, we expected participants to be more likely to endorse the indirect interpretation, and to do so more quickly, when the reply contained an emoji than when it did not contain an emoji. In addition, we examined the possibility that a reply consisting of an emoji only (i.e., with no corresponding text) might function just like a reply containing text and an emoji, as well as whether it made any difference if the emoji occurred at the beginning of the reply (Experiment 1) or at the end of the reply (Experiment 2).

## Experiment 1

The purpose of this experiment was to examine the impact of emoji on the endorsement of indirect interpretations. Participants read scenarios, questions, and indirect replies and then judged the meaning of the reply. We expected emoji to facilitate recognition of the indirect meaning (increased accuracy and decreased response time) of a reply, relative to the same reply without an emoji.

### Method

#### Participants

Participants (N = 76; 43 female, 29 male, 4 unidentified) were undergraduate students enrolled in Introductory Psychology courses who participated for partial course credit.

#### Ethics statement

The experiments presented in this manuscript were approved (exempt status) by the Institutional Review Board of Ball State University. The title of the project for which the approval was granted was "Understanding Conversations" (IRB protocol 1216210). An informed consent signature waiver was requested and granted by the IRB. Participants engaged in the consent process and provided oral consent to the experimenter.

#### Stimulus materials and design

There were 36 critical scenarios. Due to an error, one of the critical interpretations (request refusal) was positively rather than negatively worded and was dropped from all analyses, resulting in 35 critical scenarios.

Twenty-four of the scenarios (situations, questions, replies, and paraphrases) were adopted from earlier research [19,22]. Twelve new (but very similar) scenarios were written for the current research. Each scenario consisted of a brief description of two people followed by a question-reply exchange. There were three different types of situations based on the question which was either a request for an opinion (e.g., "What did you think of my presentation?"), a request for a disclosure (e.g., "How did you do in chemistry?"), or a request for action (e.g., "Can you type my term paper for me?"). There were 12 of each situation type. A sample opinion scenario is presented in Table 1 (all materials are available in the S1 Appendix). The reply always violated the relation maxim by not providing the requested information or action. These replies were all excuses and functioned by providing a reason for why the opinion or disclosure might be negative, or why one could not comply with a request. On one-third of the trials, the reply included an emoji, on one-third of the trials the reply did not contain an emoji, and on one-third of the trials the reply contained only an emoji (i.e., no words). Three stimulus lists were created so that participants saw an equal number of replies with an emoji, without an emoji, and with only an emoji, and across the experiment an equal number of participants saw each of the three versions of each reply.

**Table 1:** Sample stimulus materials: Experiment 1.

Nick and Paul are taking the same history class. Students in this class have to give a 20-min presentation to the class on some topic, Nick gave his presentation and then decided to ask Paul what he thought of it. Nick: What did you think of my presentation? (no emoji reply) Paul:/ + /It's hard / to give a good presentation. (emoji plus text reply) Paul:/ Fig C in S1 File /It's hard / to give a good presentation. (emoji alone reply) Paul:/ Fig C in S1 File ***Paraphrase*:** I didn't like your presentation.

After reading the remarks, participants judged a potential interpretation of the reply. This paraphrase was always a face-threatening interpretation of the reply (see Table 1). To keep participants from believing that the paraphrases were always reasonable interpretations, 36 filler trials were included in which the paraphrase was clearly an incorrect interpretation. In all other respects, the filler trials were similar to the critical trials: One-third of the replies included an emoji, one-third did not contain and emoji, and one-third contained only an emoji. Presentation order of the 72 scenarios was randomized for each participant.

A pretest was conducted in order to select the emoji to be used in this study. Participants (N = 41) in the pretest (who did not participate in the subsequent two main studies) were presented with 24 of the scenarios (the set adapted from [21]). Following each reply, participants were asked to choose an emoji (from 20 common emoji that were presented) that they would use to help the recipient understand the meaning of the reply. The emoji most frequently chosen for each of the three situation types was chosen for use in the present experiments. A grimace Fig C in S1 File was selected for use with the opinion and request refusal scenarios; a sad emoji Fig D in S1 File was used for the negative disclosures. Even though we used the emoji selected most often, it should be noted that, consistent with research demonstrating the underlying ambiguity of emoji [29]), consensus regarding emoji selection was relatively low. All emoji appearing in this manuscript were obtained from the Open Emoji library. Stimuli used in the experiments were Apple Emoji.

#### Procedure

The experiment was conducted on a personal computer using Eprime software. Participants first read instructions regarding the task and then engaged in four practice trials. The experimenter provided feedback regarding the procedure during these trials. Participants pushed the enter key to begin a trial. The situation description then appeared on the screen. When participants had read and understood the description, they pushed the space bar and the first remark (the question) appeared in the center of the screen. Participants then pushed the space bar when they understood the question. The reply was then presented, in chunks, in the middle of the screen. The first chunk was the replier’s name, the second chunk was either an emoji (on emoji trials) or a + (on nonemoji trials), the third chunk was the first part of the reply and the fourth chunk was the final phrase of the reply (see Table 1 and the S1 Appendix). Participants were instructed to read each chunk carefully and push the space bar as soon as they understood the speaker’s meaning. After participants indicated their understanding of the final reply chunk, a tone (500hz) sounded for 750 ms and then the interpretation of the reply was presented in the middle of the screen. Participants were instructed to read the paraphrase and decide as quickly as possible whether it was a reasonable interpretation of the preceding reply. They were instructed to push (with their right hand) the YES (1) key if it was a reasonable interpretation and the NO (3) key if it was not.

### Results and discussion

Paraphrase accuracy and paraphrase reaction time were analyzed with a linear mixed-effects model that included Situation type (disclosure vs. opinion vs. request refusal) and Emoji condition (No emoji vs. Emoji and text vs. Emoji alone) as fixed effects and the intercepts for participants and stimuli as random intercepts. Initial analyses that included random slopes for participants and stimuli failed to converge and hence were not included in the model. For paraphrase reaction times only trials for which participants endorsed the indirect reply were included. Extreme reaction times (less than 100 ms and greater than 4000ms) were treated as outliers and excluded from the analyses (< 2.6% of trials). All effects reported as significant were reliable at *p* < .05. The data used in this analysis are available at Open ICPSR. The results are presented in Table 2.

**Table 2:** Paraphrase Reaction Time (RT; in ms) and accuracy (ACC) as a function of emoji condition and situation: Experiment 1.

		Opinion		Disclosure		Request Refusal		Mean	
		RT	ACC	RT	ACC	RT	ACC	RT	ACC
No Emoji	Mean (SD)	1421^a^	.82^a^	1406^a^	.87^a^	1347^a^	.83 (.38)	1391^a^	.84^a^
		(699)	(.39)	(656)	(.34)	(612)		(656)	(.37)
Emoji & Text	Mean (SD)	1226^b^	.87^b^	1323^b^	.92^b^	1287^a^	.85 (.36)	1280^b^	.88^b^
		(590)	(34)	(549)	(.28)	(561)		(567)	(.33)
Emoji Alone	Mean (SD)	1379^a^	.87^b^	1331^b^	.95^b^	1504^b^	.80 (.40)	1399^a^	.88^b^
		(692)	(.33)	(593)	(.22)	(740)		(676)	(.33)
Mean	Mean (SD)	1341 (666)	.85 (.35)	1352 (600)	.91 (.28)	1376 (645)	.83 (.38)	1356 (636)	.86 (.34)

Means within a column without a superscript in common are significantly different at *p* < .05.

Overall accuracy was quite high (86%) but did vary over situation type, *F*(2, 2619.36) = 18.14. Most importantly, there was a significant main effect for Emoji condition, *F*(2, 2620.20) = 5.17. Post-hoc tests (Bonferroni correction) indicated that accuracy was significantly lower for replies that did not contain an emoji (*M* = 84%) than for replies that did contain an emoji, either alone (*M* = 88%) or with text (*M* = 88%). In addition, there was a significant Emoji Condition by Situation Type interaction, *F*(4, 2620.48) = 2.95. Simple effects analyses indicated that Emoji Condition was significant for opinions, *F*(2,818.18) = 3.55, and disclosures, *F*(2,827.39) = 8.98 but not for request refusals, *F*(2,827) = 1.23. For the former two situations, accuracy was significantly lower for replies without an emoji than for replies that contained an emoji (both alone and with text).

For paraphrase reaction time, there was a significant effect for Emoji Condition, *F*(2, 2170.05) = 12.38. Post-hoc tests (Bonferonni correction) indicated that reaction time was significantly faster for replies containing text and an emoji (*M* = 1280) than replies without an emoji (*M* = 1391) and emoji only replies (*M* = 1399). In addition, there was a significant Emoji Condition by Situation Type interaction, *F*(4, 2175.50) = 5.08. Simple effects analyses indicated that Emoji Condition was significant for opinions, *F*(2, 658.38) = 8.27, and request refusals, *F*(2, 653.96) = 8.95 and marginally significant for disclosures, *F*(2, 731.30) = 2.32, *p* < .10. For opinions, replies containing emoji and text were significantly faster than replies with no emoji and replies with only emoji. For request refusals, emoji alone were significantly slower than replies with no emoji and replies with emoji and text. For disclosures, replies with no emoji were significantly slower than replies with text and emoji, and marginally significantly (*p* = .10) slower than relies with emoji only.

In general, then, participants were more likely to endorse an indirect, face-threatening interpretation of a reply, and to do so more quickly, when the reply contained an emoji than when it did not contain an emoji. This effect varied over situation type, and was most clear for opinions and disclosures, and less clear for request refusals. There are differences in the pragmatic mechanisms involved in these different situation types that may account for these differences, a topic that we return to in the General Discussion.

## Experiment 2

The purpose of this experiment was to replicate Experiment 1 and to examine the possible effect of emoji location. Rather than placing the emoji before the text as in Experiment 1, in this experiment the emoji was placed after the text (for the text plus emoji condition).

### Method

#### Participants

Participants (N = 49; 7 males) were undergraduate students enrolled in Introductory Psychology courses who participated for partial course credit. None of these participants took part in Experiment 1.

#### Materials

The stimulus materials were identical to those used in Experiment 1 except that on trials containing emoji and text, the emoji was presented after the text rather than before the text.

#### Procedure

The procedure was identical to that of Experiment 1, except for the presentation of the reply which was structured as follows. For replies containing text, the first chunk was the replier’s name, the second was the first part of the reply, the third was the final phrase of the reply, and the final chunk was either an emoji (on emoji trials) or a + (on nonemoji trials). For emoji only trials, the first chunk was the repliers name and the second chunk was the emoji.

### Results

Paraphrase accuracy and paraphrase reaction time were analyzed with a linear mixed-effects model that included Situation type (disclosure vs. opinion vs. request refusal) and Emoji condition (No emoji vs. Emoji and text vs. Emoji alone) as fixed effects and the intercepts for participants and stimuli as random intercepts. Initial analyses that included random slopes for participants and stimuli failed to converge and hence were not included in the model. For paraphrase reaction times only trials for which participants endorsed the indirect reply were included. Extreme reaction times (less than 100 ms and greater than 4000ms) were treated as outliners and excluded from the analyses (less than 3% of trials). The data used in this analysis are available at Open ICPSR. The results are presented in Table 3.

**Table 3:** Paraphrase Reaction Time (RT; in ms) and accuracy (ACC) as a function of emoji condition and situation: Experiment 2.

		Opinion		Disclosure		Request Refusal		Mean	
		RT	ACC	RT	ACC	RT	ACC	RT	ACC
No Emoji	Mean (SD)	1432^a^	.85^a^	1443^a^	.91 (.28)	1298^a^	.92 (.27)	1393^a^	.90^a^
		(704)	(.36)	(632)		(456)		(610)	(.31)
Emoji & Text	Mean (SD)	1334^ab^	.91^b^	1344^a^	.93 (.26)	1318^a^	.91 (.28)	1332^ab^	.92^ab^
		(628)	(.28)	(547)		(526)		(569)	(.28)
Emoji Alone	Mean (SD)	1260^b^	.95^b^	1260^b^	.95 (.21)	1492^b^	.91 (.29)	1331^b^	.94^b^
		(615)	(.22)	(454)		(675)		(593)	(.24)
Mean	Mean (SD)	1338 (650)	.90 (.29)	1347 (551)	.93 (.25)	1370 (566)	.91 (.28)	1351 (591)	.92 (.28)

Means within a column without a superscript in common are significantly different at *p* < .05.

Overall accuracy was quite high (92%) and as in Experiment 1, there was a significant main effect for Emoji condition, *F*(2,1342.59) = 4.75. Again, accuracy was lower for the replies that did not contain an emoji (*M* = 90%) than replies with text and an emoji (*M* = 92%) and emoji only replies (*M* = 94%). However, Bonferonni post-hoc tests indicated that only the no emoji vs. emoji alone condition was significantly different. In addition, there was a significant Emoji Condition by Situation Type interaction, *F*(4, 1329.62) = 3.03. Simple effects analyses indicated that Emoji Condition was significant for opinions, *F*(2, 131.96) = 7.98, but not disclosures, *F*(2,526.23) = 1.77 or request refusals, *F*(2,478.91) = < 1. For the opinions, accuracy was significantly lower for replies without an emoji than for replies with text and an emoji and emoji only replies.

For paraphrase reaction time, there was a significant effect for Emoji Condition, *F*(2,1371.80) = 3.63. As in Experiment 1, replies without an emoji (*M* = 1393) were slower than replies containing text and an emoji (*M* = 1332) and emoji only replies (*M* = 1331). However, Bonferonni corrected post-hoc tests indicated that only the no emoji vs. emoji alone conditions were significantly different. In addition, there was a significant Emoji Condition by Situation Type interaction, *F*(4, 1398.31) = 8.52. Simple effects analyses indicated that Emoji Condition was significant for all three categories, although the pattern varied over the three categories. For opinions and disclosures, the interpretation of replies with no emoji took significantly longer than replies containing only an emoji. For request refusals, the interpretation of replies containing only emoji resulted in significantly longer reaction times than replies with no emoji and replies with emoji and text.

### Analyses of combined studies

In order to provide the most comprehensive tests of the impact of emoji on comprehension, we conducted analyses of the data from the two experiments combined. These analyses were identical to those already described except for the addition of replication as a fixed factor. The only difference between the two experiments was emoji location; for replies containing text and emoji, the emoji was presented before the text in Experiment 1, and after the text in Experiment 2. There were no significant main effects or interactions for the replication variable. The data used in this analysis are available at Open ICPSR.

**Fig 1:**
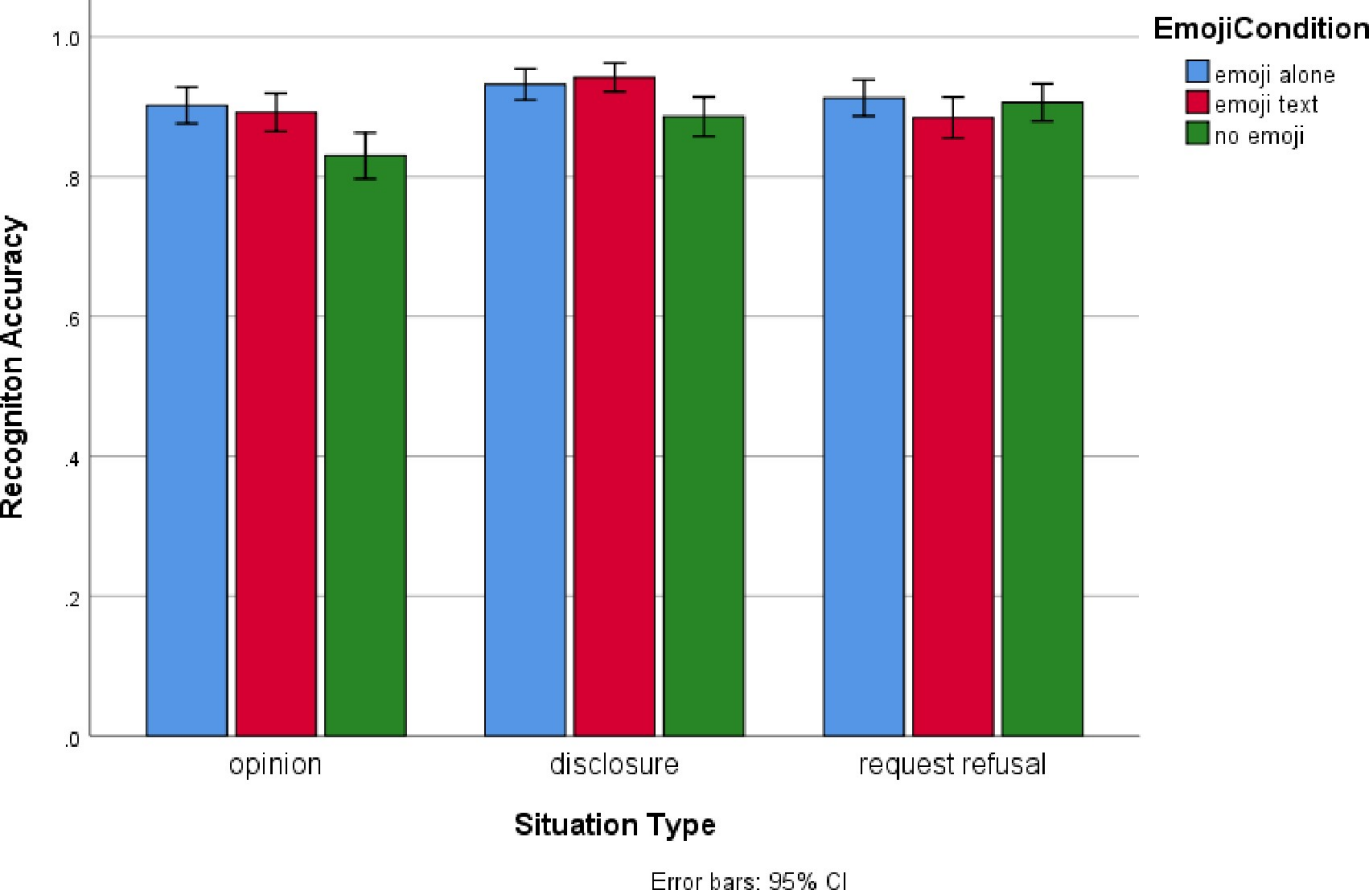
Paraphrase accuracy as a function of emoji condition and situation type (combined data).

Overall accuracy was quite high (90%) and there was a significant main effect for Emoji condition, *F*(2, 4216.70) = 10.67. Post-hoc tests (Bonferonni correction) indicated that accuracy was significantly lower for replies without an emoji (*M* = 88%) than for replies with text and an emoji (*M* = 91%) and emoji only replies (*M* = 92%). There was also a significant Emoji Condition by Situation Type interaction, *F*(4, 4216.55) = 4.21. Simple effects analyses indicated that Emoji Condition was significant for opinions, *F*(2,1373) = 9.36, and disclosure, *F*(2,3173) = 8.05 but not for request refusals, *F*(2,1259.98) = 1.49. For the former two situations, Bonferonni corrected post-hoc tests indicated that accuracy was significantly (p < .05) lower for replies without an emoji than for replies that contained an emoji (both alone and with text). The results for paraphrase accuracy are displayed in Fig 1.

**Fig 2:**
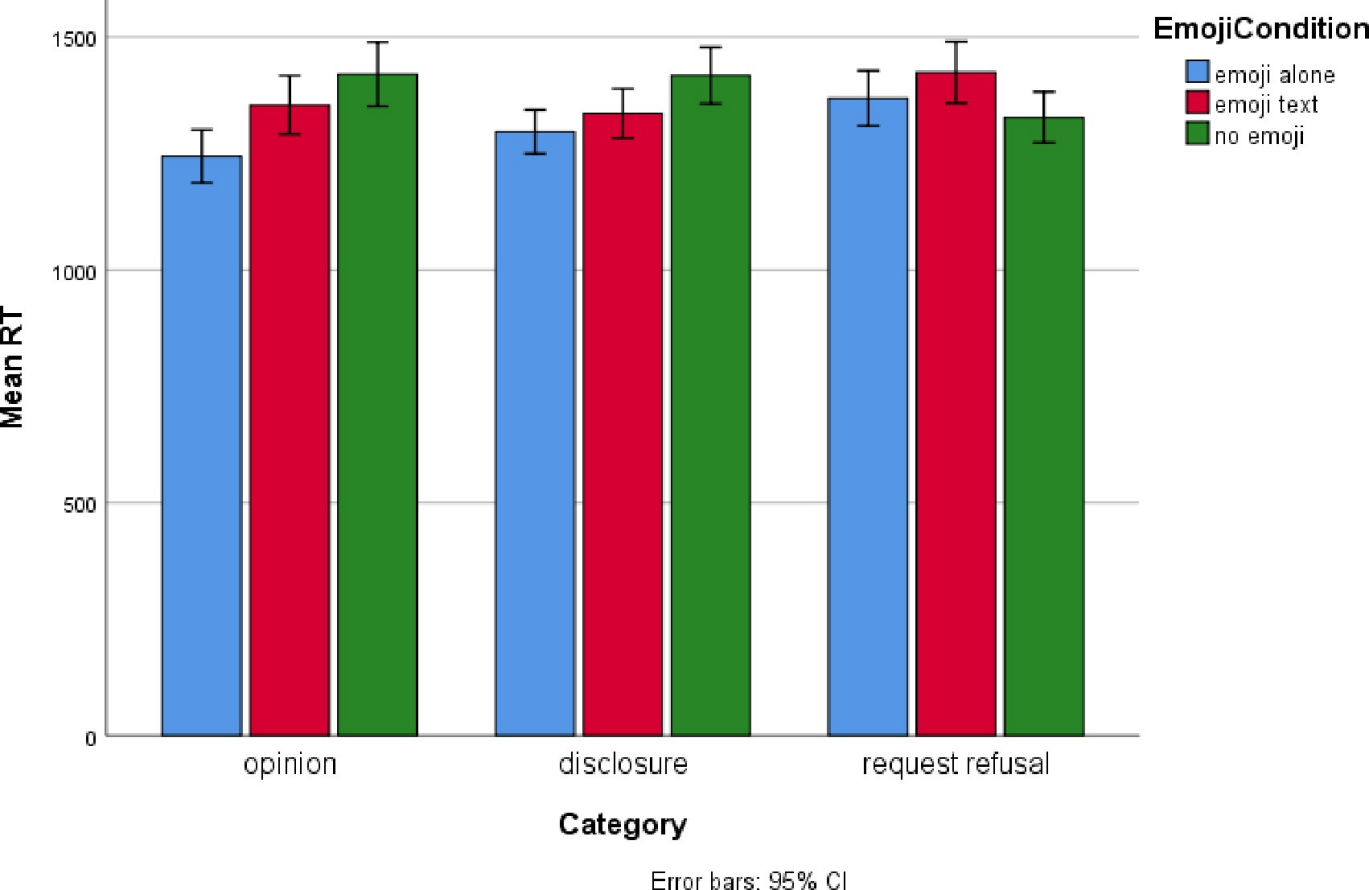
Paraphrase reaction time as a function of emoji condition and situation type (combined data).

For paraphrase reaction time, there was a significant effect for Emoji Condition, *F*(2, 3666.75) = 11.47, as well as a significant Emoji Condition by Situation Type interaction, *F*(4, 3668.14) = 6.13. Simple effects analyses indicated that Emoji Condition was significant for opinions, *F*(2, 1155.11) = 12.80 and for disclosures, *F*(2, 1225.66) = 7.06, but not for request refusals, *F*(2, 1102.05) = 2.88. For opinions and disclosures, Bonferonni corrected post-hoc tests indicated that reaction times for replies without an emoji were significantly (p < .05) slower than replies containing emoji (both alone and with text). These results are displayed in Fig 2.

## General discussion

Speakers often convey their intended meanings indirectly. Metaphors, hints, indirect requests, and so on are all examples of indirect meaning. In these instances, aspects of the context become critical for recognizing what the speaker intends to convey. Sarcasm, for example, is often recognized via the speaker’s tone of voice. In fact, sarcasm comprehension often depends less on “what” was said and more on “how” it was said [30,31]. The wealth of contextual features that are available in face-to-face communication are, of course, absent in the digital realm, and substitutes, including emoji, have emerged to aid in the communicative process.

Prior research on the communicative functions of emoji is limited. However, there has been some research demonstrating that emoji (and emoticons) can play a role in the recognition of sarcasm and irony [8,14]. In the present research we explored the role of emoji in the interpretation of potentially face-threatening, indirect replies. Indirect replies of the type examined here are ambiguous on the surface, their literal meaning violates the relevance maxim. Although such meanings appear to be routinely recognized [21], the present studies demonstrate that (some) emoji can facilitate this comprehension process. Specifically, we found that these intended, indirect meanings are recognized more often, and more quickly, when the reply contained an emoji (either alone or with text). This facilitation occurred even when there was no text, that is, an emoji alone was sufficient. Moreover, when the reply contained text and an emoji, it made no difference if the emoji was presented before or after the text, interpretation was still facilitated.

Even though comprehension was facilitated with replies containing only an emoji, as well as with replies containing both text and emoji, it is possible that the comprehension processes will be different in these two situations. In the latter situation, emoji likely function as part of the context and serve to guide the recipient to the recognition of the indirect meaning, much like facial expressions do in face-to-face interaction [1]. In contrast, when an emoji occurred without any text, the emoji itself was the reply and hence there were no words to disambiguate. This is not to say that emoji, at least those used in the current research, contain semantic content. Rather, their occurrence, combined with the lack of any words in response to the preceding question, likely prompts the recipient to infer a face-threatening meaning. Without any words in the reply, recipients must work out the intended meaning on their own, without any textual support. What this suggests, then, is that the processing of emoji may turn out to pattern like other language variables; multiple processes are likely involved in their comprehension.

Although emoji facilitated the comprehension of indirect replies for disclosures and opinions, it did not facilitate recognition of the intended meaning of request refusals. One likely reason for this difference is the role of conventionality. Indirect meanings can be conveyed in various ways, and one important dimension in this regard is conventionality. The types of indirect replies in this research were all excuses, that is, they functioned by asserting a reason for why the information was negative (for opinions and disclosures) or for why the speaker would not comply with the request (for the request refusals). There is, however, a difference between the requests for information (opinions and disclosures) and requests for action. For the latter, there is a close connection between the underlying preconditions that can be used for both performing the request indirectly and refusing the request indirectly [32]. For example, a speaker can question the hearer’s ability to perform the requested action (e.g., Can you shut the window?). These same preconditions can be denied as a means of (indirectly) refusing to comply with the request (e.g., I can’t reach it). Thus, by denying a precondition for the performance of a request, one can conventionally refuse to comply with the request. In contrast, for opinions and disclosures, there are no preconditions that could be asserted in order to convey a negative opinion or disclosure. One could refuse to provide the requested information by indicating a lack of willingness (e.g., I don’t want to tell you). But it’s not possible to convey a negative opinion or disclosure in this way. Consistent with this difference, Holtgraves [21] found that excuses serving as replies to opinions and disclosures required an inference process (recognize and reject the literal meaning) for comprehension, but excuses serving as replies to requests did not (i.e., they were recognized directly). The present results are consistent with this finding. Indirect replies that require an inference process for comprehension (replies to opinions and disclosures) are nonconventional, and hence available contextual cues can facilitate recognition of the intended meaning. These same contextual cues will have little effect in the comprehension of replies that are conventional (request refusals in this case) and hence recognized directly. Note that this pattern is similar to that reported by Filik and Thompson [15] who found that emoticons increased the likelihood of perceived sarcastic intent when the intended meaning was ambiguous, and not when there was clear contextual support for the sarcastic meaning.

Although in this research emoji facilitated recognition of intended indirect meaning for opinions and disclosures, there is no guarantee that emoji will always be facilitative. This is because the meaning of emoji is often ambiguous. For example, Miller et al [29] found that when participants rated the same emoji rendering, they disagreed on whether the conveyed sentiment was positive, neutral, or negative 25% of the time. Because of this it seems likely that emoji will sometimes hinder recognition of a sender’s intended meaning. Still, emoji are ubiquitous, and users likely believe their use facilitates communication. It may be that the successful communicative use of emoji is unique to communicative dyads. That is, over time individuals in digital contact with one another may come to use and understand the meaning of certain (perhaps idiosyncratic) emoji. Future research should be directed toward examining when emoji can hinder rather than facilitate the recognition of communicative intent.

## Supporting information

S1 File Figure guide for emojis. (DOCX)

S1 Appendix (DOCX)
