# Supplementary figures and images for: Emoji can facilitate recognition of conveyed indirect meaning
Source: PLoS One. 2020 Apr 30;15(4):e0232361. doi: 10.1371/journal.pone.0232361 (PMC7192449; doi:10.1371/journal.pone.0232361)

Figure guide (PONE-D-20-01405R1; Holtgraves & Robinson)

Figure A:
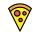


Figure B:
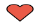


Figure C:
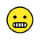


Figure D:
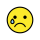

Supplement: S1 File — (DOCX) [file pone.0232361.s001.docx]
