# Supplementary material for: Emoji can facilitate recognition of conveyed indirect meaning
Source: PLoS One. 2020 Apr 30;15(4):e0232361. doi: 10.1371/journal.pone.0232361 (PMC7192449; doi:10.1371/journal.pone.0232361)
Supplement: S1 Appendix — (DOCX) [file pone.0232361.s002.docx]

Appendix

***Opinion s***

Jack is talking with Mike, an acquaintance of his. Jack gave a party last week that Mike attended. Jack wants to know what Mike thought of the party, so he decides to send a text.

Jack: Did you have fun at my party?

Mike: It's hard to throw a good party.

I didn't have fun at the party.

Nick and Paul are taking the same history class. Students in this class have to give a 20 minute presentation to the class on some topic. Nick gave his presentation and then decided to text Paul to ask what he thought of it.

Nick: What did you think of my presentation?

Paul: It's hard to give a good presentation.

I didn't like your presentation.

Martha and Sally work together in the same office. They both like to shop, especially for clothes. Martha recently bought a new coat and wants to find out what Sally thinks of it, so she decides to text Sally and ask.

Martha: What do you think of my new coat?

Sally: It's hard to find good clothes in this town.

I don't like your new coat.

Fran and Melissa are taking the same history class. Fran asked Melissa to read a draft of her term paper. Fran wants to know what Melissa thinks of her draft, so she decides to text Melissa.

Fran: What did you think of my term paper?

Melissa: That was a very difficult assignment.

I didn't like your paper.

Heather and Ann share an apartment. Recently, Heather got her hair cut and styled. She wants to find out what Ann thinks of it, so she texts her.

Heather: Do you like my new hair style?

Ann: It's hard to find a good stylist.

I don't like your new hair style.

Adam and Julie have been married for 6 months. One night Jack cooked a gourmet meal. The next day, Adam wants to know if Julie liked the meal so he texts her to ask.

Adam: How did you like the meal?

Julie: It's hard to cook a gourmet meal.

I don't like the meal.

Al is taking a painting class this semester. His friend Charles was recently visiting him. While visiting, Charles looked at Al's paintings and Al wants to know what he thinks of them. He texts Charles to ask.

Al: Did you like my paintings?

Charles: Painting with oil is very difficult.

I don't like your paintings.

Michelle bought a new book for her friend David. She wondered if he had enjoyed the book, so she decided to text him and ask how he felt about it,

Michelle: How did you like the book I bought you?

David: It's hard to write a good book.

I didn't like the book.

Jasmine and Claire had gone to the beach and Jasmine had bought a new bikini recently. Afterwards she wanted to ask Claire if she had liked the new bikini, so she decided to text her and ask.

Jasmine: Did you like my new bikini?

Claire: It's hard to find a bikini that fits your body right.

I didn't like your bikini.

Nicole has performed in a local play that her friend Ashley had attended. After the play she wanted to ask if she had enjoyed her performance.

Nicole: How did I do in the play?

Ashley: That was a very difficult role.

I didn't like your performance.

Tina had taken some family photos for her friend Elizabeth and she wondered how she liked the photos she took. She decided to text her and ask.

Tina: How did you like the photos I took?

Elizabeth: It's hard to get a good picture of my family.

I didn't like your photos.

Don was visiting his friend Dave in Atlanta. After Dave left, Dave texts Don to ask what he thought of Atlanta.

Dave: How did you like Atlanta?

Don: I think it's hectic in Atlanta.

I don't like Atlanta.

***Disclosures***

Bob and Andy are friends. Bob is taking introductory chemistry this semester and Andy wants to know how he is doing in the course, so he texts Bob:

Andy: How are you doing in chemistry?

Bob: Chemistry is a very difficult course.

I'm not doing well in chemistry.

Jim is in tenth grade and report cards were due today at school. His mom was interested in how well he did, so she sends him a text while he's at school:

Mom: How were your grades this semester?

Jim: I don't think the teacher grades fairly.

My grades aren't very good.

John hadn't seen Dennis in for a while and wanted to catch up on what was going on. They were texting each other and talking about married life. John had heard that Dennis was recently divorced and he asked him if this was true.

John: Did you just get divorced?

Dennis: I think we married too young.

I just got a divorce.

Ken and Bob are college students who know each other fairly well. Last Friday, Bob told Ken he was thinking of asking out Paula - a mutual acquaintance of theirs. Ken wonders if Paula went out with him, so he texts him to ask.

Ken: Did Paula agree to go out with you?

Bob: She's not my type.

She wouldn't go out with me.

Bill and Larry were texting after class early in the spring semester. Bill had heard that Larry was arrested for driving under the influence during finals week the previous semester. He decides to ask him about it.

Bill: Did you get arrested for driving under the influence?

Larry: It's hard not to celebrate the end of the semester.

I was arrested for driving under the influence.

Dale was texting his friend Marty. He knew Marty was on the job market and he was curious as to whether he had gotten a job.

Dale: Did you get that job you applied for?

Marty: I need to improve my interview skills.

I didn't get the job.

Sarah and Sam were old friends who were texting each other. Sarah and Sam used to work for the same company. Sarah wants to ask Sam if he has gotten a raise.

Sarah: Did you get a raise yet?

Sam: The economy is still bad.

I didn't get a raise.

Mikey had a performance with his band the night before and his friend Danny wanted to know if it had gone well. Danny decided to send him a text and ask.

Danny: How was your concert last night?

Mikey: The crowd wasn't very good.

The concert didn't go well.

Grace had heard that her son Kyle's grades were down this semester and she wanted to know if he would still be able to graduate on time. She decided to text him and ask.

Grace: Will you still be able to graduate on time?

Kyle: My classes were just harder this semester.

I won't be graduating on time.

Luke was up for a promotion at his job and his sister Lani wanted to know if he had gotten it. She decided to text him and ask.

Lani: Did you get that promotion?

Luke: My boss doesn't like me.

I didn't get the promotion.

Kayla is trying to quit smoking and her friend Vanessa wanted to know if she had. She decided to text her and ask.

Vanessa: Did you quit smoking?

Kayla: It's so hard to stop smoking.

I didn't quit smoking.

Hank and Paul are roommates. They usually play golf together on Saturday. This Saturday, however, Paul went alone. Hank wants to know how well his golf game is going, so he texts him.

Hank: How is golf going today?

Paul: I think I need glasses.

I'm not doing well at golf today.

***Request Refusal***

Barry and Al are roommates. Rent is due and Barry needs $50 to cover his share. Barry decides to text and ask Al to loan him $50.

Barry: Can you loan me $50 so I can pay the rent?

Al: I don't get paid until Friday.

I can't loan you the money.

Mark and Sam are coworkers. Mark wants to take Friday off and needs to find someone to work for him; so he decides to text and ask Sam to work for him.

Mark: Can you work my shift this Friday?

Sam: I'm having a party Friday.

I can't work for you Friday.

Jack and Frank are friends. It is finals week and they are both busy with school work. Jack finished writing his history term paper and he wants someone to read it before he turns it in. He decides to text Frank and ask if he will read the paper.

Jack: Can you read my term paper for me?

Frank: I have to study for a final tonight.

I can't read your paper.

Beth and Barbara work in the same office. Beth's car is in the repair shop and she decides to text Barbara to ask her for a ride home.

Beth: Can you give me a ride home?

Barbara: I took the bus today.

I can't give you a ride.

Frank and Mark are roommates. They usually share household chores. Frank wants to know if Mark will cook dinner tonight, so he sends him a text.

Frank: Will you cook dinner tonight?

Mark: I'm going to class tonight.

I can't cook dinner tonight.

Sandy and Cheryl are roommates. They usually share chores, and Sandy thinks it is Cheryl's turn to clean the house. She decides to text her to ask her to do it this weekend.

Sandy: Will you clean the house this weekend?

Cheryl: I'm going home Saturday.

I can't clean the house.

Beth and Susan are roommates. They are the same size and sometimes wear each others' clothes. Beth wants to know if she can borrow a blouse of Susan's, so she asks her over text.

Beth: Can I wear your silk blouse tonight?

Susan: You haven't returned the sweater that you borrowed.

You can't borrow the blouse.

The Smiths try to divide up the chores evenly. Mrs. Smith decides it's time for Tom to take out the garbage and so she texts him to asks him to do it.

Mrs. Smith: Will you please take out the garbage?

Tom: I took out the garbage last time.

I'm not taking out the garbage.

***Fillers***

Nicole had invited her friend Ashley to come to her a local play she was performing in. She decided to text her to ask if she would be able to come:

Nicole: Can you come to my play tonight?

Ashely: I don’t really like plays.

I can make it to your play.

Martha is running for city council. She texts her grandson, Jared, to ask him if he can help her campaign this weekend.

Martha: Can you help me campaign this weekend?

Jared: I have to work on Saturday.

I can help you campaign this weekend.

Bill is going on a trip this weekend and he needs to fill up his gas tank. He texts his wife, Sandy, and asks her to fill up the tank.

Bill: Will you fill up the gas tank in my car?

Sandy: I filled up the tank last time.

I will fill up the tank.

Karen's husband wants to take her on a romantic date this weekend without their kids. She decides to text her sister, Julie, and ask her to watch the kids Saturday night.

Karen: Can you watch my kids Saturday night?

Julie: I will be out of town on Saturday.

I can watch your kids.

Jared was hungry and knew his roommate was going out to eat with friends. He decided to text him and ask if he would bring him back food.

Jared: Will you bring me back something to eat?

Roommate: I don't have any extra money.

I'll bring you back some food.

Daniel was going out of town to visit his family, but didn't want to leave his dog alone for long. He decided to text his friend Alice to ask if she would watch her dog.

Daniel: Will you watch my dog this week?

Alice: Your dog doesn't like me.

I will watch your dog this week.

Taylor was driving down the road when her tire blew out. She decided to text her friend Molly to ask for help.

Taylor: Can you help me change my tire?

Molly: Changing a tire is very difficult.

I can help you change your tire.

Paul and Seth were planning to hang out at Seth's apartment, but Paul forgot he had made plans with his girlfriend. He decides to text Seth and ask if he can bring his girlfriend along.

Paul: Can I bring my girlfriend over?

Seth: There isn't much room in my apartment.

You can bring your girlfriend over.

Wallace had bought a birthday present for his brother Tom and sent it to him in the mail. He wanted to know how he liked the gift, so he decided to text him and ask.

Wallace: How did you like the gift I sent you?

Tom: It's hard to buy gifts that I like.

I liked the present.

Phil had taken his kids on a train ride and his wife Marley wanted to know if they were having a good time. She decided to text him and ask.

Marley: Are the kids having fun on the train ride?

Phil: I don't think kids really like trains anymore.

They are having fun.

Neil had been wanting to buy a new place to live so he and his sister Wanda went to an open house together. Wanda wanted to know what he thought about the house they saw so she texted him to ask.

Wanda: How did you like the house we saw?

Neil: It's hard to find a good house in that neighborhood.

I liked the house.

Drew had found a funny video on YouTube and sent it to his friend Faith. He wanted to know how she liked it so he decided to text her and ask.

Drew: How did you like the video I sent you?

Faith: It's hard to make me laugh.

I liked the video you sent.

Fred was talking to his friend Tim, who abruptly left because he and his girlfriend were having an argument. Fred wants to know what happened, so he texts Tim.

Fred: Did you and your girlfriend break up?

Tim: We are better off as just friends.

We didn't break up.

Beth knew that her friend Sam had a basketball game today and wanted to ask if his team had won the game. She decided to text her to ask.

Beth: Did your team win the game today?

Sam: I've had better games.

We won the game.

Phil's grandmother had been admitted to the hospital recently, and his friend Kurt wanted to check in to see how she had been doing. Kurt decided to send Phil a text:

Kurt: How is your grandmother doing?

Phil: Getting older can be difficult on your health.

She's doing well.

Jon knew that his friend Scott had drank a lot the night before when they went out to the bar together. He wondered if he woke up with a hangover, so he texted him the next morning to ask.

Jon: Did you wake up with a hangover?

Scott: It's been a while since I drank alcohol.

I woke up feeling fine.

The last time Sal spoke with his friend Benjamin he had mentioned that he was going to get his car fixed. Sal wanted to know if he Benjamin had gotten this done, so he texts him to ask:

Sal: Did you ever get your car fixed?

Benjamin: I've been very busy with work recently.

I got my car fixed.

Lisa knew that her sister Jessica's son was participating in a spelling bee. Lisa wondered if he had won the spelling bee, so she decided to text Jessica:

Lisa: Did your son win the spelling bee?

Jessica: Spelling can be very hard when you're young.

He won the spelling bee.

Landon knows that Jim has been struggling to keep up in math class. He decides to text Jim to see how he is doing in the class.

Landon: How are you doing in math?

Jim: Math is a tough subject.

I am doing well in math class.

Nikki and Tabby went shopping for new clothes and Tabby wanted to know how well the clothes fit on her. She decided to text her and ask.

Tabby: How did your new clothes fit?

Nikki: That store sells clothes in the wrong sizes.

The clothes fit well.

Oliver had been talking about starting to recycle and so Mitch wanted to ask if he had started. He decided to text him and ask about it.

Mitch: Did you decide to start recycling yet?

Oliver: My apartment complex doesn't have recycling.

I have started recycling.

Denise had to go to the hospital for a surgery and her father Dan wanted to ask how the surgery went. He decided to text her and ask.

Dan: How did the surgery go?

Denise: I think the doctor was new to the hospital.

The surgery went well.

Margaret and Joe live together and Joe needed to pay the rent. Margaret wanted to ask him if he was going to be able to have the money for it on time so she decided to text him and ask.

Margaret: Will you have the money for rent on time?

Joe: The landlord doesn't care when we pay the rent.

I will have the money on time.

Clyde had met his girlfriend’s parents for the first time and he wondered if he had made a good impression. He decided to text his girlfriend to ask how they felt.

Clyde: Did I make a good impression with your parents?

Girlfriend: My parents never like my boyfriends.

You made a good impression.
